
JOURNAL INFORMATION
==============================
NLM Title Abbreviation: Med Chir Rev
NLM Journal ID: 0034617

The Medico-Chirurgical Review

Publisher: Biomedical Journal Digitization Project

ARTICLE INFORMATION
==============================
PMCID: PMC4966192
PMID: 29917614
Article version: 1
Subjects: Reviews

On Fever—Eclectic or Synthetical

Print publication date: 1828 Jul 1
Volume: 9
Issue: 17
First page: 1
Last page: 20
1828 Apr 12
License: This work is free of known copyright restrictions. For more information, please see PMC Back Issue Digitization.
License URL: https://creativecommons.org/publicdomain/mark/1.0/

==============================

THE

### Medico-Chirurgical Review,

No. XVII.

[FASCICULUS VII.]

APRIL 12, 1828.

### Article I.

### FEVER.

1. *Dictionnaire de Médécine; Articles,* *Fievre—Gastro-Enterite.* M. M. Coutanceau et Rayer.
2. *A Practical Treatise on the Typhus, or Adynamic Fever.* By John Burne, M. D. &c.

It is, we think, much to be lamented, that, the noble example set by our Gallic brethren, in the publication of two successive voluminous dictionaries of medicine and its collateral sciences, should not have been followed by any similar undertaking on this side of the Channel. In a department of knowledge, as yet so essentially progressive, so mutable in its doctrines and in the manner of their application to practice, there is nothing which, in our opinion, can so effectually contribute to improvement, as short, concentrated monographs on particular diseases, frequently given to the public, in a collected and alphabetic form, as in the work, from which we select the present articles.

The usual obstacles to publication on select and favorite topics—the demand, on the part of the bookseller, for a certain quantity of matter—the apprehension, on the part of the author, that he may not be able to distend his subject to the necessary bulk, without either hiding, or disfiguring its best features, are all removed by the plan of collective labour in a dictionary, such as that now before us. Here the collaborateur furnishes a dense, perspicuous treatise, containing an epitome of every thing that has been done by others on the subject, up to the hour in which he writes, together with his own opinions, and the results of his observation and experience. The name of the writer being affixed to each article, makes him responsible to the public for his own production, and enforces, by the strongest ties we have upon mankind, their interest and fame, the necessity of making his Essay the best that he can furnish.

There is an amiable, and we conceive, an enviable peculiarity, in many of the French works of science. We often find some of their best productions under a double signature. Two friends, emulous in the pursuit of knowledge, agree to make their researches together; they assist and stimulate each other, and the result of their joint labour, is generally something superior to what either of them could have singly effected. We can boast of but few examples of this kind of literary partnership, particularly in our medical writings.

The distinguished authors of the Essays now before us, are already too well, and too favorably known to the public, to require any bibliographical notice of their works from us. M. Rayer more especially, the joint author of the first (Fièvre) and the sole author of the second article (Gastro-Enterite) at the head of this review, has given us, in his book on the Diseases of the Skin, a sufficient guarantee, as to the strength of his mind and the accuracy of his conceptions.

Although the French medical writers are generally remarkable for the precision and method, with which they treat their subjects, we cannot, we fear, hold up the article *fièvre*, as a very marked example of lucid arrangement of parts. It possesses, however, many excellencies to compensate for this deficiency. The following appear to us to be the chief objects of the first monograph.

1st. Our authors consider the *word fièvre*, with its Greek, and Latin synonymes, as a name used to represent one, or more morbid phenomena.

2ndly, They enter into "*general considerations*," on the subject of fever, and, under this head, they pass in review the opinions, as to its nature and cause, of the most celebrated pyretologists, ancient as well as modern, chiefly with a view to decide this important question, viz. whether the fevers of authors, but more particularly those of M. Pinel, ought to be considered as essential, idiopathic affections of the system, or merely as "physiological expressions of some local disease?"

3rdly, They give the conclusions to which their own researches and observation have led them with regard to this question, and for the pathology and treatment of the inflammations causing, or constituting, (in their opinions) all fevers, they refer to the distinct heads, *Enterite*, *Gastro-enterite*, *Meningite*, *&c.*

In endeavouring to open any thing like a clear path, through the dense cloud of theories that has hitherto hung over the various doctrines of fever, like the mist over the Valley of Mirza, we shall take our authors as our guides, reserving to ourselves, however, the privilege of making our own arrangements for the undertaking.

1. "The word *fièvre, fever, febris,* from *fervere, to boil*—*πυρετὸς* from *πῦρ* fire, is not, say our authors, a substantive, of which the singular is not more distinct than the plural, as some have ironically observed; it is a substantive, the plural and singular of which have been used successively in different acceptations. It is an expression which has been employed by turns to represent augmentation of animal heat—increase of heat with acceleration of the contractions of the heart—the latter phenomenon without increase of heat—a general disturbance of the functions without topical lesion—a salutary effort of Nature to cure disease, or to concoct the crude humours infecting the mass of animal fluids—a primary, and general modification of the economy, sometimes producing inflammations. An acceleration of the course of the blood, by means of a quickening of the contractions of the heart, with increase of calorification, and disturbance of the principal functions, &c."

"Fevers, according to Galen,* are diseases that come on without inflammation,

* Edit. Chart. Tom. ix. Aph. Hipp. Comment. pag. 184.

without abscess, without local pain, without erysipelas, without a special lesion of any part. 'If,' says this great physician, 'an inflammation of the side, or of the lungs produce fever, these diseases do not take the name of fever; they are called peripneumony, affection of the spleen, &c.' Celsus remarks, 'that two or three symptoms are not enough to characterize a morbid state. Heat, and increased frequency of pulsation in *the veins* are certainly the two principal characters of fever, but alone they do not constitute it, because many operations of the economy, such as fear, anger, &c. are capable of producing them.'"

M. Pinel differs but little from Galen. The word fever with him, implies increased heat, hurried circulation, disturbed functions, and the absence of local lesion. The definition given by Selle is still more vague.

"Fever is a disease variable in its course and its duration. There is cold, and heat, with the pulse sometimes more, sometimes less quick than natural. Cullen evidently follows Galen, when he defines fever as pyrexia without any primary local disease."

Thus we see, that by each succeeding definition of the word fever, the basis of a theory as to its nature and cause, nay even as to its treatment, is at once traced out.

Such being the vague and unsettled state of the notions connected with this word, we have deemed it necessary to declare, once for all, that when we may hereafter use the word fever, we would be understood to mean that state, in which we find heat of skin, quickened pulse, and deranged secretions without any reference whatever to the causes of these phenomena.

### 2. General Considerations.

There is no great difficulty in finding out to what sect our authors belong, from their readings of the great koran of physic, the works of Hippocrates. The learned Hakeem, in these, as in the book of his prophet, can find shadowed out the rudiments of all that man has ever yet discovered, as to human diseases and their cure, provided that he knows how to interpret the sacred text. In fact, there has been no view hitherto taken, either in pathology or therapeutics, of which we may not detect some traces, however obscure they may be, in the writings, authentic or apocryphal, attributed to the Father of Medicine. Thus the doctrines of the very latest and most popular living pyretologists, as to the local origin of fever, find considerable support in the recorded opinions of the Coan Sage. Take the following as an example.

"In angina, there is *fever*, pain in the head, swelling of the jaws."*

"In wounds of the brain, *fever* and bilious vomitings are the necessary consequences."†

Hence it is argued by our authors, that, as in the times of Hippocrates, the knowledge of pathological anatomy and physiology was so very limited, and as

---

* Hipp. de Morbis, lib. ii. † Hipp. de Morbis, lib. v.

there were so few opportunities of connecting the symptoms during life, with the state of the organs after death, he was induced to attach to *fever*, as the chief morbid phenomenon of every acute disease, all the other symptoms that he had observed, and thus to make it a generic term, to designate all diseases accompanied by morbid heat, but to which he could not assign a local origin, as in the cases of angina and injuries of the brain, just quoted. For our own parts, we think that there is abundant evidence to shew that the word Πυρετὸς was used by Hippocrates, on some occasions, to designate the febrile symptoms attending local inflammations which were palpable and visible to all, on other occasions, to designate the same symptoms more or less aggravated or complicated, but unaccompanied by any topical affection then cognizable. The leading groups of these symptoms, he naturally and properly divided into different types, distinguished by the modes and succession of their attacks; such as continued, intense fever—intermittent fever returning every day, every third day, &c. We think that our authors have established, in a most satisfactory manner, that the different epithets applied by Hippocrates to particular fevers, were only descriptive of symptoms accompanying that which, with him, constituted the principal phenomenon, namely, heat of skin, and not intended to designate distinct species of the same disease. Thus, fever, with the epithet *phricodes*, means febrile heat, preceded by shivering; with the epithet *lingodes*, morbid heat accompanied by hiccup; and so of the others.

We shall now endeavour to arrange under distinct heads, in a more concise manner than our authors have done, the different opinions that have hitherto been promulgated, as to the nature and cause of fever. We shall, of course, notice only the leading theories, those that appear to have given, during their respective reigns, a marked character and direction to the treatment of disease. It would be manifestly impossible to enumerate, in the space allotted to a review, all the minor opinions which physicians formed for themselves, from time to time, made up of parts selected from the greater systems, and combined according to their own views. The principal sects of pyretologists, whose theories as to the nature and proximate cause of fever, have swayed the science of medicine from its earliest records up to the present day, might, we think, be arranged under the following heads, viz. The Humorists—The Animists—The Solidists—The Essentialists—The Localists.

### THE HUMORISTS.

The earliest, beyond all question, the most universally received, the most permanent in its duration, and we might, perhaps, add, the most practically useful in its application to the treatment of disease, is that theory which was born with the healing art itself, was methodized by Galen, and is acted on by many up to the present day, we mean *the humoral doctrine.* The founder of the humoral, and, as it would seem, of many other doctrines also, was Hippocrates. He came to the conclusion, that all fevers are produced by a vitiation of the fluids. The vagueness and want of precision which we find in his writings on this subject, and his total silence as to the state of the pulse, are due to his entire ignorance of the mechanism of the circulation. He saw clearly, however, that the secreted fluids became depraved, some more palpably so than others, as soon as the fever was established. Hence his notions as to the agency of the atrabilis, &c. In short, with Hippocrates, health consisted in the existence of a perfect equilibrium in the material composition of the humors; disease in the destruction of this equilibrium; the healing art, in correcting the predominant morbific qualities of these humors.

Galen attempted to improve on these common-sense and simple notions, by attributing to each individual fluid the capability of producing a particular class of diseases; but he only succeeded in complicating them into something bordering on absurdity. The natural consequence of a theory, which supposes a separate morbific agency in each depraved fluid, was, the suggestion of distinct remedies, or purifiers, to be used as each humour became more prominently peccant, or as a particular humoral idiosyncrasy happened to prevail. Hence the almost countless multiplicity of Galenic medicines. Each fever had its distinct origin. Thus, quotidian fever was produced by the putrid pituita; tertian by the yellow, and quartan by the black bile.

These doctrines, after they had been lost in the dark ages, were again brought up by the Arabian physicians, but so mystified and complicated, as, for a time, to wear the appearance of novelty. They continued, however, to regulate the curative administrations of medicine, with the most uncontrolled sway, for many centuries.

### The Animists.

The first important schism from Galen's creed, was that of Paracelsus and Vonhelmont, the Mahomet and the Ali of physic. The former of these enthroned a demon, which he called Archæus, in the cardiac portion of the stomach, and, under his orders, he placed myriads of inferior Archæi, in the different organs of the body. All the functions were regulated by these, armed with their respective *ferments.* All diseases, and, consequently, fevers, were produced by the rebellious and improper use made by the minor Archæi of their respective ferments, and by the efforts of the great Archæus to bring them into subjection and order. Vonhelmont substituted, in his theory, the rational soul for all the Archæi, great and small. According to these theories, as each disease was looked upon as a battle, fought upon a regular plan, between the Archæus and the ferments, or between the rational soul and the morbific matter, the physician had little more to do than to look on. To these extravagant notions we owe that famous work by Stahl, "*Ars Sanandi cum expectatione.*" Hence the *médecine expectante* of the Essentialists of the late French school.

Although the archæus and the rational soul, (the *enormon* of Hippocrates and Plato revived) soon fell into disrepute, the notion, that fever is the result of a salutary effort of Nature to expel a ferment from the blood, was entertained by the great Sydenham himself, and is obscurely seen in the "*vis medicatrix*" of Cullen.

The medical theories of the Alchemists and their followers consisted in the belief of certain rude chemical actions going on amongst the fluids, and directed their efforts to the discovery of alexipharmacs, panaceas, and elixirs of longevity, to be substituted for the cumbrous farrago of Galenic simples.

The Mechanists, at the head of whom we may place Boerhaave, rejected with disdain the humoral doctrines, as far as the influence of the recrementitious fluids was concerned. They retained, however, all that was good in the views and practice of Hippocrates, and pretended to direct their own upon principles purely mechanical and mathematical. The volume and form of the globules of the circulating fluids, as compared with the capacity of the vessels containing them, formed the basis of their theories as to the cause of disease.

### THE SOLIDISTS.

Cullen may be placed at the head of these, for Hoffman was but half a Solidist. This school attributes all fevers to a spasm, or morbid contraction of the extreme capillaries. They moreover confine the term to acute diseases, unaccompanied by any primary local affection. The doubts which this theory involves, as to whether tone ought, in certain cases, to be given or taken away, naturally led to a vaccillating practice, producing little or no influence on the progress, or result of the disease. But a still more mischievous effect was produced by this doctrine. It arrested the progress of medical improvement by an artificial classification of diseases, entirely unsupported either by clinical observation, or pathological anatomy. John Hunter and his followers, when they use their favourite language, "the action of the vessels of a part," only mix up the solid parts of this theory with the immaterial vitalism of Stahl and Vonhelmont.

### THE ESSENTIALISTS,

Or, more properly, the Ontologists of Broussais, having Pinel for their chief, finding that they could not maintain the independence of all fevers upon local inflammation, limited the number to five, which they called essential, viz.

inflammatory, bilious, mucous, adynamic, and ataxic fevers. They held that the entity, or being, of which they appear to know nothing, but which they call fever, produces, by a mechanism which they do not pretend to describe, all the symptoms and organic lesions which we witness during the progress of these diseases, or find after the death of those that perish by them. This, in fact, goes to assert that fever is fever, that it will have its fling in spite of all we can do, and leads once more to the practice of the *médecine expectante* and harmless ptisans. The bitter enmity, which our authors every where manifest towards the theories of M. Pinel, renders the following homage to his candour and integrity doubly valuable:—"A faithful interpreter of facts, even when in opposition to his theoretical principles, he attributes the ataxic fever to a morbid state of the brain. This was the first step in that road, which was afterwards destined to lead to a more important reform."

### The Localists.

The gradual improvement and diffusion of anatomical knowledge, the more accurate and more frequent observation of post-mortem appearances, in fact, the general spread of science, had been long preparing the basis of a much more important theory of fever than any that had been hitherto substituted for the doctrines of Hippocrates and Galen. The Localists, or those who hold that all fevers are caused by local disease, owe the materials of their theoretical edifice to the dissections made by Bertholin, Bonetus, Morgagni, Baglivi, Chirac, Prost, the Hunters, Bichat, and a host of others. The traces of inflammation or congestion, almost always found about the encephalon and abdominal viscera of those that perish from fever, very early induced different pathological anatomists to consider one or other of the varieties of these local affections as the proximate cause of fever. Chirac conceived that fever is owing to inflammation of the brain. Baglivi says, that the fevers called malignant are produced by phlegmon, or erysipelas of the intestines. M. Broussais, however, appears to have been the first who digested the facts collected up to his day, as to local irritations and inflammations, particularly those of the stomach and bowels, into a regular code of doctrinal pathology. Our authors, zealous supporters of his doctrine in all its ramifications, thus introduce this celebrated pyretologist to our notice.

"Penetrated by the sublime views of Bichat as to the sympathies, rich in numerous facts observed with a rare sagacity, M. Broussais came to overturn, from the very foundation, the antique edifice of fevers. In his works, as well as in his lectures, he has applied himself, for many years, to demonstrate, that the fevers which had been called essential, were nothing more than local diseases, inflammations, nay, even *gastro-enterites*. The following propositions (say our authors) may be looked upon as the summary expression of his doctrines.

"1mo. Fever, considered in a general and abstract manner, is never other than the result of a primitive or sympathetic irritation of the heart, through the effect of which this viscus hurries its contractions.

"2do. Every irritation, sufficiently intense to produce fever, is an inflammation.

"3tio. All the fevers of authors are connected with gastro-enteritis, simple or complicated. This they have all overlooked when unaccompanied by local pain, and even when accompanied by pain, they consider it as an accident. Authors have sometimes said, that certain fevers depended on inflammation of the digestive organs, but they have never said that these fevers, the pretended essential fevers, cannot have any other cause; never that they were produced by the same mechanism as the fever of peripneumony; never, in short, that there are no essential fevers.

"4to. It is by gastro-enteritis that small-pox begins; by gastro-enteritis and an acute occular, nasal, guttural, or bronchial catarrh, that measles and scarlatina commence.

"5to. Intermittent and remittent fevers are periodical *gastro-enterites*, but the encephalon and other viscera are irritated sympathetically, as in continued fevers, and may also become the principal seat of irritation, and put on inflammation in an acute or periodic manner.

"6to. The fevers termed pernicious differ from other fevers, only by the violence and danger of congestions."

M. Broussais holds that these propositions are proved by the following facts; we give his own words.

"1mo. All the causes of fevers act locally; they all irritate the gastric mucous membrane, that point of the organism on which the action of every morbific cause strikes.

"2do. In almost all fevers there are unequivocal local symptoms of irritation of the stomach and small intestines, a fact that does not allow us to overlook the existence of gastro-enteritis.

"3tio. The sympathetic symptoms, in the absence of symptoms of gastric irritation, demonstrate evidently, though indirectly, the existence of gastro-enteritis in all fevers.

"4to. A great number of organs do not participate in a morbid state in fevers, and of those that do, some are more, some less affected by it.

"5to. The adynamic and ataxic symptoms are due to irritation.

"6to. After death we always find traces of gastro-enteritis.

The above assertions constitute the doctrinal code of the Broussaists, certainly the most important medical sect of the present day.

The curative administrations in all fevers, practised and inculcated by this, as, by all other sects, are the legitimate offspring of their theory, as to the proximate cause of the disease.

We do not mean to notice here, the minor varieties of Localists of our own country, (schismatics from this parent stock,) neither those who found their innocent little heresies on particular symptoms and traces of organic lesion, not very frequently present; nor those, whose vague notions as to topical congestions, have led to destructive, and perhaps, erroneous practice.

We pass over in silence the individual examination of M. Pinel's five essential fevers, and the arguments adduced to prove, that they are but so many examples of gastro-enteritis. Indeed, our authors can see nothing but their favourite "*gastro-enterite*" in the putrid fevers of Pringle, the typhoid epidemics, described by Fracastorius, Tissot, Roederer, &c.; nay, the very intestinal worms, often found after death, are, according to M. Broussais, produced by *gastro-enterite.*

Severe and peremptory as the Animists were in their condemnation of the doctrines of their predecessors, the Humorists—the Solidists in their enmity towards the Animists; we shall find that the Localists are equally, if not more determined in their condemnation of their immediate masters and rivals, the Essentialists. The following ultimate, and sweeping conclusion, to which our authors arrive, respecting the theories of the Pinel-school, will, we think, justify this assertion.

"We conclude from the researches which we have made preparatory to writing this article, and from the facts which we have accumulated in its different paragraphs,—that the general descriptions of inflammatory, bilious, mucous, adynamic, and ataxic fevers, arising from an attempt to reconcile facts dissimilar, or as yet undetermined, being inexact, arbitrary, and false, ought never hereafter to be reproduced in works of pathology."

As the size and expense of the work, from which we have selected the present article for review, must render it inaccessible to many of our readers, we have taken much pains to offer a concentrated view of the different theories, in the examination of which, our authors have given proofs of great research and critical acumen. We have been more particular in our notice of the doctrines of M. Broussais, because they exercise, at present, a paramount influence on the whole medical practice of the Continent; and, because they are rapidly gaining ground in this country. Theories are important, not so much from the reality and consistency, or utter groundlessness of the facts, upon which they appear to repose; as from the decided bent they have at all times given, when adopted generally, to the measures by which we endeavour to prevent, or modify disease. We have followed our authors in their theoretical research, in order that we might arrive, with minds better prepared than they otherwise could have been, to the discussion of the practical parts of our subject, the anatomical characters, the symptoms, and treatment of these inflammations, which, by some, are thought to cause, or constitute fever; by others, to be but secondary effects of a more general cause. Be this, however, as it may, it is not the less true, that if we can remove the phlegmasiæ, or congestions alluded to, we shall very rarely fail in curing the general functional disorder, by which they are always accompanied.

### Gastrite, Gastro-Enterite, &c.

As the admiration which our authors, (particularly M. Rayer,) every where express, for the doctrines and practice of M. Broussais, appears to us to border, rather too closely, on enthusiasm, we have deemed it prudent to take in a little practical ballast from M. Chomel, on the subject of *gastritis*, before we approach the stormy ocean of *gastro-enterite*.

"Gastritis, or inflammation of the stomach," says this enlightened physician and laborious observer, "is one of those affections, which, for some years past, have particularly engaged the attention of medical men, and given rise to a great number of works, for the most part polemic. If discussions were of a nature to throw much light on the history of disease, our knowledge of the one in question would have made rapid progress. But the experience of ages has proved, that the sciences of observation march with facts; and that scholastic disputes have seldom profited them much. Instead of collecting with care, and without any other intention than that of coming at the truth, numerous examples of this disease, the greater number of physicians have devoted themselves to theoretic discussions, have almost entirely neglected to collect facts, or have viewed them only through the dangerous prism of prejudice. What has been the result? Inflammation of the stomach, embarrassed by an almost infinite number of useless writings, obscured by inexact, or truncated observations, is at this moment, one of the phlegmasiæ, of the symptoms and anatomical characters of which, we have but an imperfect knowledge. This assertion may appear false, to some, but will not surprise those who have carefully watched the sick, and opened many bodies. In fact, if on the one hand, it is often impossible during life, to determine with certainty, what the state of the stomach will be after death; and if, on the other hand, the anatomical examination of this viscus is not generally sufficient to determine with precision, whether there were, or were not symptoms of gastric inflammation during life; are we not compelled to acknowledge, that this phlegmasia is one of those, our knowledge of which, is as yet but very imperfect. The number, and the discrepancy of the writings, of which this disease has been the subject for many years, would be almost enough to demonstrate this assertion.

"We do not, however," continues M. Chomel, "pretend to say, that gastritis is at all times, and in all its degrees, an obscure affection; we shall see on the contrary, that in many of its forms, it is marked by symptoms as defined as those of pleurisy and pneumonia."

### GASTRO-ENTERITE.

We pass over the remainder of M. Chomel's excellent monograph on gastritis, for the purpose of entering at once upon the legitimate domain of M. Broussais.

Nothing can be more lucid than the order, nothing, in our opinion, better judged, than the method, observed by M. Rayer, in his distribution of the heads, or points of view, under which he has treated this celebrated disease, or rather this *panto-pathological* essence of all diseases. The whole article is a perfect model of what a medical monograph ought to be. There is neither the laboured verbiage of speculative theory, nor the fatiguing minuteness of narrow observation. There is enough, however, from which to stock the mind with the most useful parts of what is known on this interesting subject, and enough to stimulate it to further enquiry. In short, we conceive it to be the very best, and most practically useful essay, that we have as yet seen to emanate from the school of Broussais.

The following paragraph contains some statistical details curiously indicative of the influence of medical theories.

"Gastro-enteritis—a name proposed by M. Broussais, to designate the simultaneous or successive inflammation of the stomach and small intestines, is, of all the phlegmasiæ the most frequent, and, at the same time, that which has been oftenest overlooked or mistaken. You will not find it indicated in any nosological table. Gastritis itself was not long ago pretty generally looked upon as a very rare disease; for of twenty-eight thousand, two hundred, and ninety-nine sick admitted into the civil hospitals of Paris, in 1807, *six only* were designated in the definitive returns, as labouring under inflammation of the stomach, whilst six thousand, one hundred, and forty-three, were treated for continued or remittent fevers. Such a result cannot be comprehended, except by admitting that, according to Brown's principles, the name gastritis was only given to inflammations of the stomach, produced by poisons; or, perhaps it may be better to suppose with Hufeland, and many French physicians, that, in consequence of some occult changes having taken place in the constitution of the atmosphere, the bilious, mucous, and adynamic fevers have been supplanted in these latter times, by gastro-intestinal phlegmasiæ, the frequent appearance of which is now no longer disputed.

M. Rayer, after shewing that the specific and separate denominations, *gastritis* and *enteritis*, have been often vaguely applied by nosologists, in their artificial arrangements, to the simultaneous, or immediately successive inflammations of the stomach and small intestines, observes—

"That as by far the best authenticated part of the history of the phlegmasiæ of the digestive organs are the changes they undergo, it must be by these changes that their inflammations can be best characterised and specified." "I am (says our author) certain, that I shall be able to render more intelligible the variety of phenomena produced by these inflammations, by beginning the description of gastro-enteritis with the alterations that constitute it."

Previously to a separate enumeration of the various alterations occurring in gastro-enteritis, our author makes the general remark, that—

"All the forms, and all the terminations of acute and chronic inflammation, observable on the external teguments, or on the different points of the gastro-pulmonary mucous membrane, may take place in the stomach and small intestines, but with unequal frequency. These gastro-enteritic phlegmasiæ, noticed by Morgagni, Prost, &c. and very recently pointed out by M. Broussais, have furnished materials to Andral, Bichat, and Louis, for novel and important observations."

According to his proposed plan, M. Rayer commences with the organic alterations, (of which he describes twenty-one,) that have been found in the stomach and bowels of those who are supposed to have died whilst labouring under gastro-enteritis. The changes that occur in the colour of the mucous membrane are first considered.

*Accidental red tints*, which he compares with cutaneous exanthems, are said to be the most constant of all the effects of this phlegmasia. They appear either under the form of *vascular arborescence*, or of circumscribed patches, varying from the most intense red to the deepest brown. Sometimes there are a number of little red spots, like the marks in purpura, occasioned by minute but separate submucous hæmorrhages.

All these changes of colour are most frequently found at the bottom, and great curvature of the stomach and on the intestinal valvules. An universal deep violet tint of the mucous membrane, black ecchymosed spots, and sometimes effusions of blood into the cavity of the stomach, may, we are assured by Morgagni, be produced by the course of the blood in the vena portæ being obstructed. The slate-coloured grey, the greenish-grey shades, which M. Broussais has sometimes remarked, affecting the gastro-intestinal mucous membrane, he considers as products of inflammation; nay, the morbid dead-white paleness of the coats of the stomach and small intestines, noticed by Bonetus, but more particularly remarked by M. Guersent, as frequently found in children, is set down to the account of inflammation by our author; as also the softening, to the consistence almost of boiled starch, described by this writer.

Here we would take the liberty of remarking, that if every shade of colour, from the darkest red to the deadliest white, may give proof of inflammation having existed in the tissue where they are found, we would say, "*nimium ne crede colori.* The necessity of this caution we have seen frequently exemplified by that accurate pathologist Laennec. In his post-mortem examinations, he would order a portion of a healthy artery, say of the aorta, to be detached, and filled with the gore of the subject. At his next clinical lecture he would order this preparation to be exhibited, when the lining membrane of the artery was always found stained or dyed a lively red, such as could not at the moment be either rubbed or washed off.

*Softening (ramollissement)* of the stomach and small intestines; "very rare (says our author) in the fœtus and the old—frequent in new-born infants and at the period of weaning—often observed in adults, more particularly in lying-in women. This disease attacks indifferently both sexes." By John Hunter it was attributed to the solvent action of the gastric juice; but is now pretty generally allowed to be the result of inflammation. This view seems to be confirmed by the pustules and figured inflammatory patches that generally co-exist with this singular affection, and by the success of antiphlogistic means in its treatment.

> "This disease (says M. Chomel) is always accompanied by acute pain in the stomach. To this organ the patient refers all his sufferings. Can we then, hereafter, assert, that the mucous membrane of the stomach is insensible? that it expresses its sufferings only by sympathetic phenomena? Is it not, on the contrary, evident, that it obeys the common law, and indicates its lesions, like other organs, by pain, and the disorder of its functions?"

We would strongly recommend to our readers the recent work of M. Louis on this very interesting subject.

*Ulcerations*—a frequent termination of inflammation in most parts of the body, and certainly the most unequivocal testimony, that the tissues in which we find it after death had been inflamed during life. The greater frequency of ulcerations in the lower portion of the ileum is attributable to the greater frequency of pustulous, inflammation in that part of the intestine, and not, as has been asserted by some writers, to the greater abundance of mucous follicles. "The duodenum, so plentifully furnished with follicles, is, of all parts of the small intestines, the least liable to ulceration."

*Gangrene* is an exceedingly rare termination of gastro-enteritic inflammation, notwithstanding M. Broussais' assertion, that he had often observed it. Our limits will not allow us to do more than merely to enumerate the other alterations which pathological anatomy is said to have found in this disease. These are thinning of the parietes of the intestinal canal—thickening of ditto—diminution, or augmentation of caliber—pustules—vesicules—papules—membraniform exudations—cream-like exudations—inflammation of the submucous cellular tissue—emphysema—œdema—perforations—vegetations—schirrous and medullary matter—tubercles, &c.

The first stage of gastro-enteritis consists in a mere sanguineous injection of the mucous membrane. The second in any of the various morbid exudations—the third in ulcerations—the fourth in thickening, or thinning of the coats, and the formation of accidental tissues. The fifth, in gangrene or destruction of the parietes themselves. As the first, second, third, and fifth stages may be developed within the first four days, they are said to belong to acute gastro-enteritis. The fourth stage, or deep and permanent alterations of texture, belongs exclusively to chronic gastro-enteritis.

*Acute Gastro-enteritis.* All ages and sexes, from the fœtus in utero to the centenarian, are liable to this disease; and every thing that can be introduced into the stomach, from the viands of the alderman's feast to the most acrid poison, from tonic draughts and emetics to knives swallowed whole, may produce it. Starving itself, a principal remedy for this disease, when carried to excess, gives rise, according to J. Hunter, Dumas and Magendie, to inflammation, and even ulceration of the stomach.

"But of all the causes of this malady, the inflammation of other organs is the most remarkable, and certainly the least noticed. Dupuytren, Abernethy, and Broussais have successively pointed out the influence of large burns, inflamed wounds, &c. in exciting gastro-enteritic inflammation."

*Symptoms.* Of the four local phenomena usually assigned to inflammation, viz. pain, heat, redness, and swelling, only the first two are appreciable during life in this disease; and they are both generally in proportion to the intensity of the inflammation, except when complicated with cerebral affections, and then they are never so sensibly felt. Pressure on the region of the epigastrium seldom, or never fails to cause pain, when gastro-enteritis is present, but we should take care in applying it, not to mistake what may be the effect of our own exertion, or the patient's natural sensibility, for the morbid tenderness of an inflamed organ. This particular phlegmasia we may say, is never found uncombined with other important derangements, as has been satisfactorily proved by the dissection-returns drawn up at the Hôtel Dieu, by order of M. Dupuytren.

According to the school of Broussais, it is to the various combinations, or groupings of the symptoms attendant upon this disease, in its different stages and complications, that we owe the countless variety of fevers described by authors.* We cannot help acknowledging that there is much truth in this observation. When the constitutional symptoms common to all inflammations were present, with a white tongue and viscid saliva, 'twas the mucous fever, with a yellow tongue, the bilious, with a black, the putrid fever. When there was tenderness of the abdomen, with much debility, it was the adynamic fever. When the gastro-enteritic inflammation was complicated with cerebral symptoms, it was the ataxic; with thoracic symptoms, it was the mucous catarrhal fever, or the pneumonia notha.

If it be true, and we believe it is as good as proved, that all the symptoms known to accompany European fevers at least, also accompany the disease we are treating of, in its acute form—if it be true that in nine-tenths of these fevers there are evident signs of gastro-enteritic inflammation during life, and that unequivocal traces of its having existed are found after death—if it be true, that this connexion of fever with the phlogosed state of the digestive organs was not efficiently noticed before the time of Broussais, then must it be also true, that this pathologist has conferred an incalculable benefit on the healing art, by calling the attention of those that exercise it, to this important, and hitherto unknown coincidence.

It has been proved beyond all contradiction, by the most ample statistic records of post mortem examinations at the Hôtel Dieu—by the experiments of Orfila, by Abernethy, and many other authorities, that large inflamed wounds, burns on the trunk or extremities and poisons applied to the surface, produce inflammation of the gastro-intestinal membrane, of the thoracic viscera, of the encephalon. The successive effects of this transition of inflammation, whether it be by sympathy, irritation, or absorption, from the surface to the centre are, an arrest, or alteration of chylification, depraved secretions, tenderness of the epigastrium, nausea, quickened circulation, heat, loss of appetite, emaciation, and all the complicated derangement of function, that extensive organic lesion never fails to bring with it. From these symptoms, as they may be variously combined in different subjects, under different circumstances, the closet-nosologist, or speculative essentialist, can compose any variety of fever, and establish an ideal species according as he finds mucus, bile, debility, or delirium, prevail. But practical anatomy acknowleges only one disease, made up of certain phenomena consecutive upon a burn, a wound, or a capital operation.

* Sauvages gives 150 varieties of fever.

*Treatment.* In these cases, at least, there is a cause of fever anterior to gastro-enteritis. Broussais says that it is an irritation; but in his second proposition he states, that "every irritation that produces fever is an inflammation." See the effect of this theoretical hitch upon his practice. He applies leeches in every variety of fever, in all its stages, nay almost in every disease, to the region of the stomach, the thorax, the head, the extremities; pursuing the sympathetic irritation, or inflammation of *gastro-enterite* through all these different localities; and this, to the almost total exclusion of every other remedial measure, except the most rigid starvation, which he calls "diete absolue."

The above comprises the whole treatment of acute *gastro-enterite*, and consequently of every variety of continued fever. The expulsion of foreign bodies from the stomach and bowels, the recalling local inflammations that may have suddenly disappeared, or the subduing those that appear to keep up the disease by sympathy, can scarcely constitute an exception.* In the following paragraph on prophylactic treatment, M. Rayer offers some good, though perhaps not very palatable advice to old gentlemen, and bold practitioners.

"This disease will become more rare in old men, when they shall have given up their elixirs of longevity, their stomachic tinctures and pills—their precautionary purgatives. It will become less frequent with all classes of society, when physicians, more reserved in the employment of potent medicines, shall have begun to count a little less on the tolerance of the stomach."

Emetics, purges, (amongst the rest, calomel,) nay, even bark, are all formally denounced. Nothing, as we said before, is left with which to combat disease, by this exclusive theory of localism, but leeches and starving. This dangerously simplified practice is not the result of, though its utility is said to be confirmed by, experience. It is the direct consequence of the third article of the Broussais-creed, as already quoted, viz: "that all fevers are connected with, and referrible to, gastro-enteritis."

### Contrast of Localism with Humorism.

How much more natural, how much more consistent with the facts, of which we are best assured in pathology and therapeutics, is the notion, that some change must take place in the circulating fluids before fever can be developed? The practice that results from this theory rejects none of the *juvantia*, overlooks none of the *lædentia*. It leaves their management where it ought to be left, to the practised judgment of the competent physician. All that we have hitherto

* A costive state of the bowels persisting, without any alvine discharge during five, or even ten days, is looked upon as a good symptom, and is not interfered with by M. Broussais.

learnt by the study of symptoms and signs; all that we have been taught by the mechanical analysis after death, of the organs that appeared to suffer during life, confirm the justness of this notion, and contribute to its development. Do we not observe the fluids become depraved as the constitution becomes more generally, and more intensely involved in fever? Do we not see all the organs resume their healthy functions, as the blood throws off by sweat, by hæmorrhage, by urine, or by stool, something, which a moment before formed a part of its own stream? But the very source of that stream is in the stomach and bowels. The principle that regulates the proportion, the quality, the relative position of its molecules, is the nervous energy. That which affords due excitement to this regulating power, is the healthy blood. No man will pretend to say, that there can be fever when all the fluids are in their healthy condition, because, on the one hand, there is due nourishment of the nervous mass, on the other, due distribution of its influence. But, as the paramount laws of matter, generally, cannot be sacrificed to individual preservation, the very organs, whose office it is to prepare and convey the materials of health to the different parts of the animal machine, must, from their very structure, take up and prepare poisons under certain circumstances, or produce by their influence, when morbidly excited, deleterious changes of combination.

Every thing then that we do in disease, ought to tend towards re-establishing the purity of the fluids when it has been disturbed. The practice of the Father of Physic was founded upon this principle. He purged upwards and downwards, to clean and prepare the digestive organs, when they appeared to suffer from foulness. He produced direct diminution of the circulating fluids by bleeding. He administered demulcents, refrigerants, corroborants. He attended to diet, and encouraged critical discharges. But above all, he distinguished the cases and the stages of disease, in which the adoption of these respective plans of treatment might be advisable. He practised an art that required the highest powers of the mind for its successful exercise. The Broussaists, on the contrary, like the Brownists, have reduced the whole science to one or two unbending maxims, which they call physiological, to be learnt in an hour, and practised without distinction; viz: that all fevers consist in *gastro-enteritis*, and their cure in local bleeding and starving. These remedies, excellent on some occasions, we have seen carried to such indiscriminate, and destructive lengths, by M. Broussais himself, that the subjects of them perished, perfect bloodless skeletons; and, instead of shewing signs of an inflammation requiring such rigour, the whole alimentary canal resembled the finest oiled paper, in paleness and transparency; exhibiting perhaps, a few arborescent patches of red, at the most declining and sharp folds of the small intestines.

There is yet another disadvantage in this doctrine, besides those already noticed. Its views do not extend beyond the limits of anatomy, and this has been lately brought to a pitch of advancement, beyond which we cannot hope to see it carried, without drawing largely on the imagination. This doctrine does not direct our research towards that other great opening that leads to chemical pathology; a source from which, we venture to predict, the next great light will illuminate our science. This is an almost uncultivated field, and in the present state of our knowledge, holds out the most brilliant hopes to the zealous and qualified labourer. We should rejoice to see such men as Prout and Orfila, enter with energy upon the track of organic chemistry.

Broussais, his followers, and his opponents, have done much good. To their controversies we owe the rapid progress that has been made in the pathological anatomy of the hollow viscera—the prudent caution now observed, even in this country, in the use of drastic purges and emetics. But from Broussais alone, we have learned the full value of that symptom, so important in fever, the sensibility of the epigastrium to external pressure. It is by applying to the etiology of this, and its subordinate functional derangements, the lights of morbid anatomy, that we are sometimes enabled to guide our practice in the commencement of fevers, so as, by prudent depletory, evacuant, and dietetic measures, to shorten their course, and mitigate, or prevent their worst symptoms.

The pathology and treatment of chronic gastro-enteritis, as understood by M. Rayer, do not come within our plan. They are not necessarily connected with fever. We, therefore, pass them over, as well as the heads, *intermittent*, and *epidemic gastro-enteritis*, because they would lead too far into polemic theory, and because the reader is referred to other articles of the dictionary, such as *fièvres intermittentes*, *typhus*, *&c.* The very titles, however, with what we have already said, will afford a tolerably correct notion of the almost unlimited extent, to which Broussais and his disciples apply their pathological pass-word. We must, however, add, that those who neglect to make themselves acquainted with the morbid alterations, the symptoms, and the principles generally, upon which the doctrines of this school are founded, want, in our opinion, a large portion of that knowledge, which a good physician of the present day, ought to possess.

We shall close this article with the concluding words of M. Rayer.

"The alterations of the stomach and intestines are so varied, and so few comparative therapeutic experiments, have been, as yet, made under well determined circumstances, that it would be difficult to ascertain, even approximately, the sum of the cures which it is permitted to hope for from the antiphlogistic plan, as compared with the more active measures, such as moxas, blisters, setons, &c. In fine, if in the present day, we see but few practitioners administering medicines for the cure of *particular symptoms*, as was, heretofore, the case—if physicians no longer blindly combat *pains in the stomach* with narcotics—*dyspepsia* with bitters, elixirs, mineral-waters, &c.—*sour regurgitations* and *belchings* with absorbents—*vomitings* by anti-emetic potions—*constipation* by calomel—*diarrhœa* by astringents, it is because, since the splendid researches of Pujol, and of Broussais, it is generally acknowledged, that the inflammatory alterations which produce these different symptoms, ought alone to form the source of curative indication."

### 2. *A Practical Treatise on the Typhus or Adynamic Fever.* By JOHN BURNE, M.D. &c. &c.

WE had already closed the *Dictionnaire*, and were about to say, in the shape of a postscript, that, though our French *confreres* might have written the article on *typhus*, to which they refer, we should be compelled to put off the consideration of it to a future opportunity, the editors not having yet given it to the world. Under these circumstances, nothing could be more *àpropos* than the appearance of Dr. Burne's book. Instead, therefore, of waiting for our neighbours' monograph, which, in all probability, is still in the bosom of fate, we shall endeavour to give our readers a brief sketch of the opinions and practice advocated by the ingenious author of the work before us.

Dr. Burne divides all continued fevers into two species—*inflammatory* and *adynamic*. The basis of this division is the presence, or the absence, of "depression of the nervous energy." Both these species may, or may not, be accompanied by local inflammations. They are both, however, thus robbed of every thing like anatomical characters. The word *adynamic* defines itself. In fact it may be said to be attended by a "*non possum*" in its fullest acceptation. The principal symptoms of "four degrees" of this fever are given, and, strange to say, the stomach is not once alluded to in their description; nor is the state of this organ once mentioned in the account of post-mortem appearances.

Pinel, who gave to this disease "a habitation and a name," but whom our author does not notice, divides typhus into *two stages*,* (and we think he is borne out by nature) viz. the inflammatory and the nervous. Amongst the symptoms of the first stage, are "*nausea* and *vomiting*"—in the second stage, "pains when the belly is touched, owing to an *inflamed state of the intestines*, which is never entirely wanting, for traces of it are *always found in the dead body*."

"*Seat and Nature of Adynamic Fever—Primary Cause.*" In reply to the questions suggested by these headings, our author (at p. 161) comes to the following conclusions, after having commented rather sharply on some of the modern theories.

"1mo. That the adynamic fever has no local seat.

"2do. That its nature is a morbid condition of the blood, produced by the operation of the primary cause, the respiration of a contaminated or poisoned atmosphere.

"3tio. That this blood, acting on the brain and nervous system, is of itself sufficient, in very many instances, to bring about the deranged functions that constitute the phenomena of the adynamic fever."

After what we have already said in our strictures on the doctrines of M. Broussais, we need not add any further comments on the views which these most important propositions lead to. Suffice it to say, for the present, that, if Dr.

---

* Nosograph. Philosoph. tom. i. p. 148, 6me. edit.

Burne's proposition stood thus, we should immediately subscribe to it. *That a morbid condition of the blood, however produced, is sufficient, in all instances, to account for the phenomena of fever.*

Organic chemistry being likely, ere long, to excite the attention of pathologists in no ordinary degree, we think it right to mention here, that, according to our author, (p. 142) "blood drawn while the adynamic fever is urgent, is surcharged with carbon." According to Dr. Clanny, of Sunderland, there is no "free carbonic acid" in typhous blood. The latter grounds his opinion on his own experiments.

"*Pathology of the Adynamic Fever.*" Under this head, in Chap. VII. come the "morbid appearances," which the great Laennec would have called the anatomical characters, and placed in Chap. I. We strongly recommend to our readers, the remarks of the author upon the effects of opening the thorax and great vessels of the heart, before the head is examined, and vice versâ, on the appearance of the vessels of the brain, and the entrance of air into them.

The inflammatory alterations found in the intestines, as well as tympanitis, when it occurs, are considered as the direct results of the congestion of fæcal matter, and "putrid decomposed intestinal secretions lying in the most depending portions of the canal," and are said to be "an effect, not a cause, of fever." The only proofs, however, brought forward, that the phlogosis of the mucous membrane is a secondary, and not a primary affection in typhus, are the cases of those who, during the progress of their disease, shewed no symptoms of enteritis, until the fever was fully formed, and who ultimately recovered.

"*Treatment—Bloodletting.* It is in these cases," says our author, "in which the adynamic fever is quickly developed, that its course may be frequently shortened by the abstraction of blood: but it must not be supposed that blood-letting will *always* cut short an adynamic fever, as it does an inflammation, as a pneumonia. No such thing."

In this remark we entirely agree.

As every practical writer recommends a favorite remedy, so Dr. Burne is loud in his praises of *mercurial inunction*, though he condemns calomel and blue pill, as "*griping*, *irritating*," &c.

"Mercurial friction of half a drachm of the unguent. hyd. fort. twice a day, has produced more rapid and favorable changes, in very severe cases of adynamic fever, than any other remedy I have ever seen administered."

As to the *quomodo* and the *quando*, it may be rubbed on any part of the body.

"When the brain is much affected, when there is inflammatory action going on —and, indeed, in all cases, and at all times, when the return of the secretions is obstinately protracted."

We have seen this remedy extensively employed in epidemic fever, of a typhoid character, and we thought with advantage, but we have also seen it produce unequivocal mischief, as the very best remedies will do, when pushed to imprudent lengths by men, who ambition the character of bold practitioners.

The reader will find recommended in Dr. Burne's book a well-timed use of aperients and enemata, an attentive observation of the colour and quality of the discharges—a strict attention to diet—a meeting of indications as they arise, not upon any insulated or empirical principles, but upon the broad basis of the views that he has taken of the nature and seat of the disease. In short, we consider this book as affording a good example of the superior prudence and efficiency of "a mixed treatment," guided by observation, and based upon a rational humoral pathology, as compared with the generalizing compendious practice of intolerant localists. The work before us, though a very useful addition, as it stands, to our stock of practical knowledge on the subject of typhus, would be at least a more agreeable one, if its pages were occasionally relieved by a little shading from either ancient, modern, or cotemporary authorities. Had Dr. B. not disdained to read, or, having read, to quote, he would not have omitted to notice the eruptive disease* peculiar to the adynamic patient, between the second and the seventh day—the anatomical characters of *petechiæ* as differing from the typhoid exanthem†—the tendency to various kinds of natural crisis, &c. We conceive it to be our duty to deprecate this autocratic, exclusive plan of writing upon a subject, which has been so ably investigated by so many great men—this setting us down to a solitary meal, however nutritious, when so many instructive guests might have been invited to meet us. If an author be either too lazy, or too proud, to strengthen his own conclusions, by an opposition to, or a comparison with, those of others; his readers will try to invalidate them by this very process, and will often succeed. To him who reads, all written authorities are of equal weight, except where the author is personally known. Notwithstanding these errors of omission, we can safely recommend Dr. Burne's book to the favourable notice of such of our readers, as may be disposed to take a plain, common sense, useful view of continued fever.

The late period at which Dr. Burne's work appeared (after the construction of this article)—the hurried glance which we have been obliged to take of it—and other considerations unnecessary to be stated—leave us the option (and we shall shortly have the opportunity) of again reverting to certain doctrines and opinions in the publication, when noticing the section on fever in Dr. Bright's work, of which one more analytical article yet remains for insertion in the course of the present number.

---

### II.

### *On Functional Affections of the Heart and Arteries.* M. Laennec.

In our last number we noticed the subject of neuralgia of the heart, and also angina pectoris. We shall now advert to some other nervous affections of this organ.

#### Palpitation.

Every beating of the heart which is sensible and unpleasant to the individual, and, at the same time, more frequent than natural, is termed palpitation. The

* Vide Thucidides, Raikem, and Bianchi, Boin, Pinel, &c.
† Rouchoux.
